# Coauthor Country Affiliations in International Collaborative Research Funded by the US National Institutes of Health, 2009 to 2017

**DOI:** 10.1001/jamanetworkopen.2019.15989

**Published:** 2019-11-22

**Authors:** Joshua C. Grubbs, Roger I. Glass, Peter H. Kilmarx

**Affiliations:** 1 Fogarty International Center, National Institutes of Health, Bethesda, Maryland

## Abstract

**Importance:**

The US National Institutes of Health (NIH) is the largest funder of biomedical and behavioral research in the world. International collaborative research—a subset of NIH’s portfolio—is critical to furthering the agency’s health research mission.

**Objective:**

To quantify the extent of the NIH’s international collaborations and the relative importance of this research through the lens of publications.

**Design, Setting, and Participants:**

This cross-sectional study used bibliometric data from the Web of Science database to analyze trends in the growth of NIH-funded publications from January 1, 2009, to December 31, 2017, and examined their importance using national affiliations of all coauthors listed, h indices, and citation impact scores. All countries with coauthor affiliations in NIH-funded publications during the study period were included. Data were analyzed from October 22 through November 16, 2018.

**Exposures:**

Country affiliations of coauthors’ institutions in NIH-funded publications indexed in the Web of Science database from 2009 to 2017.

**Main Outcomes and Measures:**

Trends in the number of NIH-supported publications with non-US coauthors during a 9-year period and their relative importance assessed by h index per country and category-normalized citation impact (CNCI) for groups of country affiliations in 2017.

**Results:**

From 2009 to 2017, the annual count of NIH-funded publications increased 46.2% from 67 041 to 98 002. This increase was driven in part by an increase in publications with a non-US author alone or as a collaborator with a US author compared with those exclusively with US authors, reflected by an increase in the percentage of publications with non-US coauthors from 28.3% to 34.8%. Moreover, in 2017, publications coauthored by US-affiliated and non–US-affiliated investigators had a higher mean CNCI (1.99) than those whose authors were only US affiliated (1.54) or non–US affiliated (1.35). China became the most frequent publishing partner, with 6982 coauthored publications and the greatest increase over time among non-US countries.

**Conclusions and Relevance:**

In a 9-year period when the NIH budget remained relatively unchanged, an increase in the number of publications occurred with a growing trend toward more international collaborations of authorship; these publications also had a higher CNCI than publications with only US or only non-US authors. The findings suggest that international collaboration is a vital and growing component of the NIH’s research output and likely reflects increased globalization of biomedical research.

## Key Points

**Question:**

What are the trends, scope, and importance of US National Institutes of Health–funded publications with international collaboration from 2009 to 2017?

**Findings:**

In this cross-sectional bibliometric analysis, National Institutes of Health–funded publications indexed in the Web of Science database increased by 46.2%, and the percentage of those with at least 1 coauthor with a non-US affiliation increased from 28.3% to 34.8% from 2009 to 2017. Publications with US-affiliated and non–US-affiliated coauthors had a greater mean citation impact score than those with exclusively US or non-US authors.

**Meaning:**

International collaboration in National Institutes of Health–funded research is a critical, growing, and high-importance component of the National Institutes of Health’s biomedical and behavioral research mission, contributing to its overall productivity.

## Teaser

This cross-sectional study uses bibliometric analysis to quantify the extent and relevance of international research collaborations funded by the US National Institutes of Health.

## Introduction

The US National Institutes of Health (NIH) is the largest funder of biomedical research in the world.^1,2^ During fiscal year 2019, the NIH used its $39 billion budget to support scientific research in pursuit of improving human health and basic science.^3^ The NIH funds research at non-US and domestic institutions; much non–US-affiliated research is conducted with funding awarded to US investigators who collaborate with non–US-affiliated scientists or trainees.^4^ The NIH recognizes that science knows no borders and values the contributions of international collaboration for quality biomedical research. Supporting projects that bring together people and institutions from across the globe provides critical opportunities to advance science, identify new diseases with unique populations, and develop better diagnostics, treatments, and cures.^5,6,7^ Clinical trials for infectious diseases, such as human immunodeficiency virus, Ebola virus, dengue, and Zika virus, for instance, require scientists in the United States to work with investigators in affected regions. Particular scientific opportunities that benefit researchers around the world may exist in isolation, such as differences in the response to chemotherapy for breast cancer based on country of origin and genetic background; high prevalence of unique, single-gene alterations that predispose a localized population in Colombia to hereditary, early-onset Alzheimer disease; or interventions to reduce arsenic exposure and its consequences in Bangladesh.^8,9,10,11^ Rapid advancements in communication technology and the trend toward team-based research have facilitated such international collaborations, allowing scientists to freely exchange data and ideas.^12,13^

To assess the importance of international research at the NIH through one lens of scientific productivity—peer-reviewed publications—we evaluated the extent of NIH-funded international collaborative research and characterized trends during the past 9 years. Bibliometric analysis offers a powerful tool to better understand this output of research and provides an alternative to other traditional methods for portfolio analysis.^14,15,16,17^ By comparing trends in the number of publications over time and a metric of scores, such as the h index, researchers can describe the relative importance of publications with coauthors affiliated with different countries. To answer larger questions about the importance of US and non-US coauthorship overall, the category-normalized citation impact (CNCI; a normalized metric that describes how frequently an article has been cited compared to other articles in its discipline)^18^ adds another dimension to the analysis.

We have conducted a bibliometric study to assess the growth of NIH-funded publications and characterize the scope of international collaboration supported by the NIH during a 9-year period when the NIH budget increased from approximately $30 billion to $35 billion but experienced an inflation-adjusted decrease.^19^ We evaluated the CNCI and the number of publications overall as a function of coauthors’ country affiliations to achieve these goals. Insights into the global reach of NIH funding is critical to understand the current research landscape and inform future investments. Collaborating institutions, including those from academia, government, and the private sector, can use these findings to guide their own decision-making to build productive international research partnerships.

## Methods

The Web of Science (WOS; Clarivate Analytics) database was queried for NIH-funded publications using a comprehensive Boolean search string of the full names and acronyms of all NIH institutes and centers in the “funding text” field. We limited our analyses to the 9-year period from January 1, 2009, to December 31, 2017, because WOS only began indexing funding data in 2008, and records for 2018 were incomplete at the time of this analysis.^20^ The WOS “analyze results” feature was used to sort records by countries or regions and download aggregate counts of NIH-funded publications by country affiliation of coauthors. Countries were categorized by income level according to World Bank country and lending groups.^21^ Additional search strings were used to query the WOS database for publications by institutes and centers and create reports on coauthors’ national affiliations as described above, using 2017 as the sample year. Publications with funding from multiple institutes and centers were included in each relevant report. InCites (Clarivate Analytics), a WOS tool, was used to calculate the CNCI for all NIH-funded publications in 2017 grouped by coauthors’ national affiliations (US coauthors only, non-US coauthors only, or US and non-US coauthors).^18^ The CNCI used baseline citation rates by WOS subject area centered around a score of 1.00, with higher scores representing greater importance. All statistics were descriptive and presented counts and proportions of publications in the sample without reference to population parameters. The Strengthening the Reporting of Observational Studies in Epidemiology (STROBE) reporting guideline was used in the reporting of this cross-sectional study. This analysis of publicly available publication data did not constitute human subjects research and did not require approval by an institutional review board or informed consent.

Data were analyzed from October 22 through November 16, 2018. The citation report feature in WOS was used to calculate 1-year h indices for each country affiliated with NIH-funded publications in 2017. The h index is a metric that reflects the number of publications and citation counts in a single number.^22^ For example, an author’s h index would be 20 if he or she had published at least 20 articles that had been cited at least 20 times each. This measure can also be applied to institutions or countries.^22,23,24^ Previous bibliometric studies have found 1-year h indices to be highly correlated with those from larger citation windows, making the statistic a valid tool for comparison.^25,26^ The data were limited to publications in 2017 to match the time frame for annual publication volume. Countries were ranked by number of publications and h index in 2017. Finally, records for all NIH-funded publications from 2009 to 2017 were sorted by citation count.

## Results

**Figure 1.**
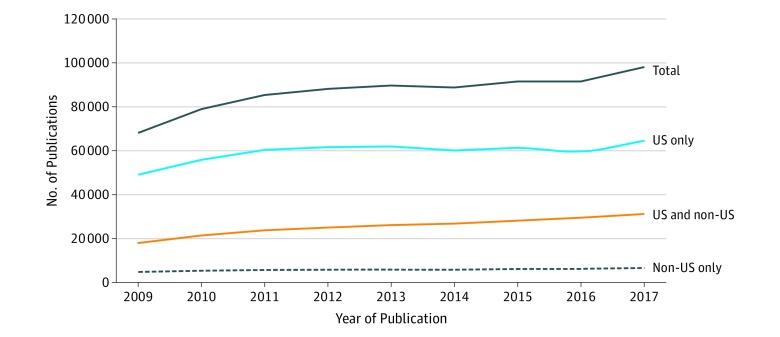
Annual National Institutes of Health–Funded Publications by Country of Authors’ Affiliations, 2009 to 2017

From 2009 to 2017, the number of NIH-funded publications increased 46.2% from 67 041 to 98 002 (Figure 1). The greatest percentage increases were in publications with US-affiliated and non-US-affiliated authors (increase of 13 392 [82.7%]) compared with those exclusively with non–US-affiliated authors (increase of 1702 [61.5%]). Publications with US-affiliated coauthors alone increased more slowly at a rate of 33.0% (n = 15 863).

As a result of the differential growth rates, the proportions of each group shifted between 2009 and 2017. The fraction of studies with US and non-US coauthors increased from 24.1% (16 188 of 67 041) to 30.2% (29 580 of 98 002), whereas those exclusively with non-US coauthors increased from 4.1% (2773 of 67 041) to 4.6% (4479 of 98 002). Consequently, the overall percentage of publications with non–US-affiliated coauthors increased from 28.3% (18 961 of 67 041) to 34.8% (34 059 of 98 002). Publications with solely US coauthors accounted for 71.7% (48 080 of 67 041) in 2009 but decreased to 65.2% (63 943 of 98 002) in 2017.

**Figure 2.**
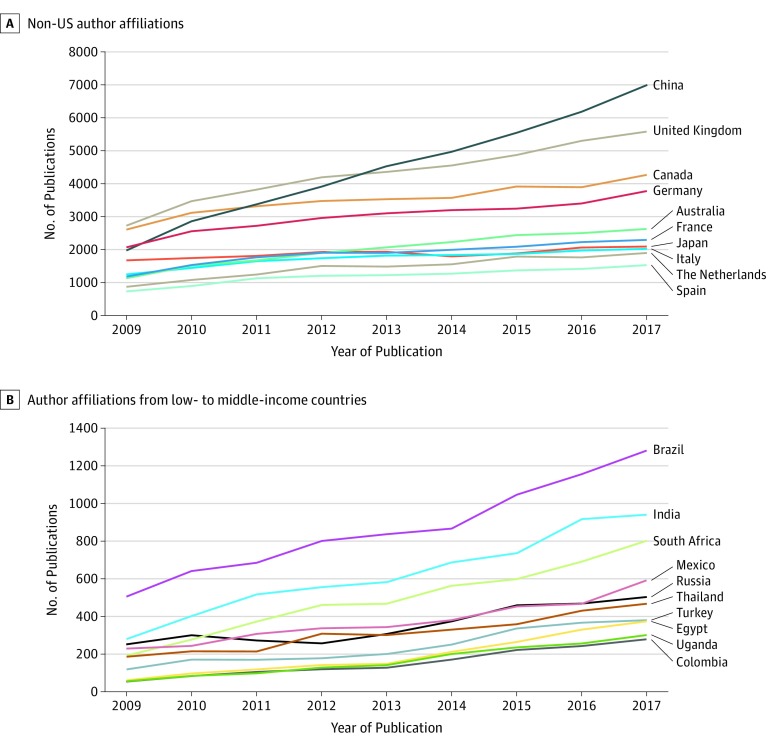
Annual National Institutes of Health–Funded Publications for 2009 to 2017 A, Top 10 non–US author affiliations. B, Top 10 low- and middle-income country author affiliations (excluding China).

In 2017, the coauthors most represented in NIH-funded publications were from China, the United Kingdom, Canada, and Germany (Figure 2A). Although all countries experienced increases in their annual publication rates, the greatest increases were from publications with coauthors from China (from 1976 to 6982; 253.3%), the United Kingdom (from 2733 to 5582; 104.2%), Canada (from 2614 to 4260; 63.0%), and Germany (from 2059 to 3780; 83.6%). China was the most frequent country affiliated with publications that included US and non-US authors (n = 6982). With the exception of China, the numbers of authors from low- and middle-income countries (LMICs) all experienced sustained lower growth in the same time (Figure 2B).

**Figure 3.**
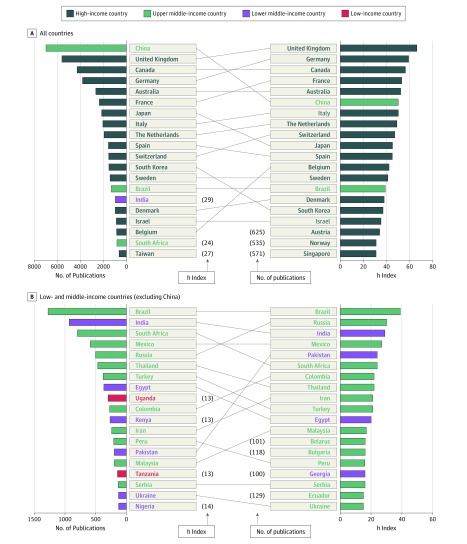
National Institutes of Health–Funded Publications and h Indices for 2017 A, Top 20 countries of authors’ affiliations. B, Top 20 low- and middle-income countries of authors’ affiliations, excluding China (6982 affiliations; h index, 50).

To examine our most significant global partnerships in research, we identified the country affiliations most represented among NIH-funded publications in 2017. These were ranked by the total number of publications and their importance as measured by the h index (Figure 3). Of the top 20 most represented country affiliations, 16 were classified by the World Bank as high-income countries (HICs), 3 as upper middle-income countries, 1 as a lower middle-income country, and none as low-income countries. Of the top 20 LMIC affiliations, 12 were upper middle-income countries, 6 were lower middle-income countries, and 2 were low-income countries (Uganda [301 publications] and Tanzania [151 publications]). Using the h index as the primary metric for ranking the importance of a country’s publications, however, elevated the United Kingdom (h index, 66) and Germany (h index, 59) to the top of the list (Figure 3A). Canada (h index, 56) remained third from the top, and China (h index, 50) moved down to the sixth place by h index. India, South Africa, and Taiwan no longer appeared among the top 20 countries, replaced by Austria (h index, 34), Norway (h index, 31), and Singapore (h index, 31). In other words, the latter countries’ publications had a greater importance by this metric despite totaling fewer publications overall.

The same analysis was repeated using only LMICs (Figure 3B). The h indices for China and Brazil were 50 and 39, respectively, ranking them as the top 2 LMICs by publication output and h index in 2017. Uganda and Tanzania, the only 2 low-income countries ranked among the top 20 LMICs for publication output, did not fall among the top 20 countries by h index. Similarly, Nigeria did not rank among the top 20 countries in the same group. Russia (h index, 30), Colombia (h index, 22), Iran (h index, 21), and Malaysia (h index, 17) ranked substantially higher in the list by h index.

Of the top 20 countries ranked by h index, 12 were European countries and 4 were high-income East Asian and Pacific countries as classified by the World Bank.^21^ Internationally collaborative publications tend to be most productive and have greater h indices and CNCI among European partners and Canada as well as various high-income Asian countries and China. China stood out for its most rapid growth in the same period and as the only LMIC among the top 10 represented countries. Marked increases have occurred in NIH-funded publications among LMICs, especially in Africa and Latin America, although these rates still lag behind rates of the HICs.

The top 10 most cited NIH-funded publications with 1 or more non–US-affiliated coauthors from 2009 to 2017 all included at least 1 US-affiliated coauthor (Table).^27,28,29,30,31,32,33,34,35,36^ Japan and the United Kingdom were the most represented country affiliations (3 publications); followed by China, Germany, Saudi Arabia, Sweden, and Switzerland (2 publications); and then France, Israel, and Singapore (1 publication). Publication topics primarily covered genomics experimental methods and computational biology.

**Table.** Top 10 Most Cited National Institutes of Health–Funded Publications With Non-US Coauthors, 2009-2017

Rank	Source	Country Affiliations^a^	No. of Times Cited
**All Countries**			
1	Hanahan and Weinberg,^27^ 2011	Switzerland	34 286
2	Tamura et al,^28^ 2011	Japan	30 593
3	Tamura et al,^29^ 2013	Japan, Saudi Arabia	16 292
4	Adams et al,^30^ 2010	United Kingdom	9737
5	Li et al,^31^ 2009	China, United Kingdom	8672
6	Schindelin et al,^32^ 2012	France, Germany, and Switzerland	7834
7	Ronquist et al,^33^ 2012	Sweden	6743
8	ENCODE Project Consortium,^34^ 2012	China, Germany, Japan, Spain, Singapore, Switzerland, and United Kingdom	6020
9	Kumar et al,^35^ 2016	Japan, Saudi Arabia	5702
10	Grabherr et al,^36^ 2011	Israel, Sweden	5342
**LMIC Only**			
1	Genomes Project Consortium,^37^ 2015	Bangladesh, China, Colombia, Gambia, Jamaica, Mexico, Nigeria, Pakistan, Peru, Sierra Leone, Turkey, Vietnam, and 14 HICs (13)	2278
2	Uhlén et al,^38^ 2015	India and 3 HICs (11)	1868
3	Vos et al,^39^ 2015	Afghanistan, Bangladesh, Belize, Benin, Brazil, Bulgaria, China, Colombia, Côte d'Ivoire, Egypt, Ethiopia, Fiji, Gambia, Georgia, India, Indonesia, Iran, Iraq, Jordan, Kenya, Lebanon, Malaysia, Mexico, Morocco, Nigeria, Pakistan, Papua New Guinea, Philippines, Romania, Russia, Rwanda, Serbia, South Africa, Sudan, Syria, Tanzania, Tunisia, Turkey, Uganda, Ukraine, Vietnam, Zambia, and 32 HICs (31)	1565
4	Roadmap Epigenomics Consortium,^40^ 2015	China and 4 HICs (2)	1220
5	GTEx Consortium,^41^ 2015	Brazil and 8 HICs (2)	1171
6	Global Burden of Disease Cancer Collaboration,^42^ 2015	Brazil, China, Colombia, Ethiopia, Georgia, India, Iran, Malaysia, Mexico, Nigeria, Philippines, Russia, South Africa, Vietnam, and 17 HICs (23)	933
7	INSIGHT START Study Group,^43^ 2015	Brazil, South Africa, Thailand, Uganda, and 7 HICs (25)	896
8	Heneka et al,^44^ 2015	China and 11 HICs (3)	880
9	Chang et al,^45^ 2015	China and 2 HICs (10)	795
10	Shao et al,^46^ 2015	China and 8 HICs (5)	793

Abbreviations: HIC, high-income-country; LMIC, low- and middle-income country.

^a^ All publications except for that of Uhlén et al^38^ included at least 1 US-affiliated coauthor. Data in parentheses indicate percentage of authors from LMICs.

Publications with LMIC-affiliated coauthors (with or without US coauthors) had much fewer citations (Table).^37,38,39,40,41,42,43,44,45,46^ Most publications were studies of genomics and the global burden of disease, drawing from LMICs and HICs alike. However, all first authors of the top 10 most cited NIH-funded publications in 2017 were affiliated with HICs. The percentage of LMIC-affiliated authors ranged from 2% (Roadmap Epigenomics Consortium^40^; GTEx Consortium^41^) to 31% (Vos et al^39^), with a median of 11.5% in this sample. Only 1 publication (Uhlén et al^38^) did not include a US-affiliated coauthor.

For 2017, we analyzed NIH-funded publications among the 27 institutes and centers at the NIH. Of the 98 002 publications in 2017 that reported funding from the NIH, 42 872 (43.7%) did not indicate which institute or center provided support, and 16 788 (30.5%) included funding from more than 1 institute or center and are counted multiple times. The National Cancer Institute was most often acknowledged for US-affiliated publications (7407 [62.06%]) and publications with non–US-affiliated authors (4505 [37.8%]), followed by the National Institute of Allergy and Infectious Diseases (2315 [45.7%]) and the National Heart, Lung, and Blood Institute (1840 [31.5%]). The Fogarty International Center had the highest percentage of non–US-affiliated publications in 2017 (855 [90.0%]), followed by the National Institute of Allergy and Infectious Diseases (2315 [45.7%]), the National Human Genome Research Institute (421 [44.0%]), and the National Institute of Biomedical Imaging and Bioengineering (551 [43.0%]). All of the institutes and centers at the NIH were represented among the set of publications in 2017 with at least 1 non-US coauthor.

Publications with US and non-US coauthors had the greatest importance as measured by CNCI (1.99), greater than that of publications with only US coauthors (1.54) or those with only non–US co-authors (1.35). The baseline CNCI rate for all NIH-funded publications in the same year was 1.65, placing reports by US-only and non–US-only coauthors below the mean in the sample.

## Discussion

From 2009 to 2017, a period when the NIH budget was relatively unchanged, the number of NIH-funded publications nonetheless grew by 46.2%, reflecting a global trend in biomedical research. Much of this growth was attributable to the more rapid increase in the number and percentage of publications with non-US coauthors. Furthermore, publications with US- and non–US-affiliated coauthors had greater importance as measured by the CNCI than publications with only US- or only non–US-affiliated authors. Notably, NIH director Francis Collins, MD, PhD, has consistently voiced his support for greater international collaboration, and all 27 institutes were represented with publications having coauthors.^47^

These results reinforce earlier findings that internationally collaborative publications tend to be cited more frequently.^48,49^ However, the rankings by the CNCI in this study are important for 2 additional reasons: (1) they suggest that the trend for greater importance among internationally collaborative papers can be extended to the country level for biomedical research as a whole rather than by institute or subdiscipline within a country, as is already more thoroughly investigated^5,12,50,51,52,53^; and (2) the CNCI also follows the same broad pattern when applied to publications associated with a given funding source. Bibliometric analysis provides another element for portfolio analysis that can be used to summarize the global expansion of biomedical research.

Of note, the most cited reports primarily covered genomics, methods, and the global burden of disease. The first 2 topics listed rank high among countries regardless of income, whereas burden of disease studies were most common when the scope is limited to LMIC-affiliated publications. Even among these, the proportion of LMIC-affiliated coauthors did not exceed 31%, suggesting that HICs largely drive the research. Considering the large number of collaborators needed to collect enough data for these investigations, collaboration itself was likely a mediator for the enhanced visibility and high CNCI of these publications.

The predominant country affiliation for non-US authorship is China when ranked by volume, CNCI, and h index. The dramatic increase in coauthored publications may be the result of increased bilateral programs for postdoctorate trainees and visiting fellows, but a rapid survey of the data does not suggest postdoctorate trainees and visiting fellows are the sole contributors to research. Sampled articles listing Chinese-affiliated coauthors generally described equitable distribution of authorship responsibilities when such statements were present.

### Limitations

This analysis has several potential shortcomings inherent to the methods used. Databases such as WOS do not index all publications and thus cannot provide a complete data set for bibliometric analysis.^14,54^ However, of the various databases available, WOS captures the most publications in biomedical research with full funding information (29%) and accordingly was used for this study.^54^ Furthermore, publication data, including funding sources and coauthor country affiliations for each study, can be missing or inaccurate if entered into the database incorrectly.^14,55^ All authors in the database had country affiliations listed, but publications not captured by the database could nevertheless limit the internal and external validity of the study. The metrics themselves—h index in particular—must be interpreted in context and with the recognition that the country of publication can influence these statistics.^14,24^ The analysis also does not account for the balance or authorship order of US-affiliated and non–US-affiliated coauthors on a given report. Finally, coauthors’ contributions do not factor into the analysis, eliminating any potential differences among the distribution of those who write the manuscripts, for instance. The distribution of coauthors’ roles by country and country income merits further research. In addition, national affiliation does not necessarily imply different geographical locations for coauthors. International fellows working at NIH-funded facilities in the United States still qualify that publication as an international collaboration if those coauthors maintained their affiliations with non-US institutions. Future research on this topic should consider investigating the different roles and breakdown of authorship by country affiliation. Previous studies have addressed some of these concerns but often on the institutional level rather than on a global scale. Developing a more robust profile of NIH-funded work at this level may help scientists and policy makers support important biomedical research.

## Conclusions

International collaborative research offers investigators new opportunities to maximize importance and interact with non-US investigators by sharing opportunities from populations having unique features and genetics, exposures, or health care delivery and bringing new technologies to emerging or existing health problems. As biomedical research continues to grow, scientists will need to work together across borders to solve increasingly complex health issues. Improving human health across the globe depends on it.
